# The Second Life of *Citrus* Fruit Waste: A Valuable Source of Bioactive Compounds †

**DOI:** 10.3390/molecules26195991

**Published:** 2021-10-02

**Authors:** Caterina Russo, Alessandro Maugeri, Giovanni Enrico Lombardo, Laura Musumeci, Davide Barreca, Antonio Rapisarda, Santa Cirmi, Michele Navarra

**Affiliations:** 1 Department of Chemical, Biological, Pharmaceutical and Environmental Sciences, University of Messina, 98166 Messina, Italy; carusso@unime.it (C.R.); amaugeri@unime.it (A.M.); gelombardo@unime.it (G.E.L.); laura.musumeci@unime.it (L.M.); davide.barreca@unime.it (D.B.); antonio.rapisarda@unime.it (A.R.); 2 Fondazione “Prof. Antonio Imbesi”, 98123 Messina, Italy; 3 Department of Pharmacy-Drug Sciences, University of Bari “Aldo Moro”, 70125 Bari, Italy

**Keywords:** *Citrus*, waste, circular economy, flavonoids, polyphenols, nutraceuticals, by-products, valorization, phytochemicals

## Abstract

*Citrus* fruits (CF) are among the most widely cultivated fruit crops throughout the world and their production is constantly increasing along with consumers’ demand. Therefore, huge amounts of waste are annually generated through CF processing, causing high costs for their disposal, as well as environmental and human health damage, if inappropriately performed. According to the most recent indications of an economic, environmental and pharmaceutical nature, CF processing residues must be transformed from a waste to be disposed to a valuable resource to be reused. Based on a circular economy model, CF residues (i.e., seeds, exhausted peel, pressed pulp, secondary juice and leaves) have increasingly been re-evaluated to also obtain, but not limited to, valuable compounds to be employed in the food, packaging, cosmetic and pharmaceutical industries. However, the use of CF by-products is still limited because of their underestimated nutritional and economic value, hence more awareness and knowledge are needed to overcome traditional approaches for their disposal. This review summarizes recent evidence on the pharmacological potential of CF waste to support the switch towards a more environmentally sustainable society.

## 1. Introduction

In the last decades, the enhancement of life quality brought an unceasing growth of the worldwide population, leading to an excessive consumption of resources and, consequently, to a considerable production of waste. The term “waste” refers to something unused or not used to full advantage. Therefore, high waste generation may result from inefficient management of its disposal. This process implies high costs and may be the cause of both ecosystem (i.e., soil and water) and living being health damage, because waste is released into landfills or burned to produce energy. Therefore, a recycling/re-use perspective is necessary to allow the reduction of the extent of produced waste and related socio-economic costs [1]. This could mitigate the excessive exploitation of raw materials and allow taking advantage of the potential economic and biological value of the waste. In this regard, European Commission suggested to “improve economic performance while reducing pressure on natural resources through efficient use of them” [2]. Recently, the management of fruit and vegetable by-products moved from a linear economy to a more sustainable circular one [3]. Traditional linear economy follows the “take-make-dispose” model by which raw materials are recovered, then processed into products that are consumed until being disposed as waste [4]. Circular economy aims at recovering valuable materials directly from waste to enter a new production cycle and to minimize its generation [5], therefore following the “take-make-use” model [4]. The pillars of this novel approach are “recover, recycling, repurpose, remanufacture, refurbish, repair, re-use, reduce, rethink and refuse” [6]. Similarly, the “green chemistry” approach aims at reducing waste generation, despite it was primarily conceived to limit the threats to both environment and human health by employing renewable energetic sources, non-toxic reagents, and more ecological processes (i.e., air pressure and room temperature) [7]. Moreover, according to circular economy, another consequence of revalued waste is the potential recovery of compounds endowed of notable biological value [8].

Fruits and vegetables are among the most consumed foods worldwide. Indeed, a large amount of waste results from both consumption of fresh fruit and industrial processing to produce juices, concentrates, jams, marmalades, jellies, canned fruit, dehydrated products, flavoring agents, beverages and healthy drinks [3]. *Citrus* fruits (CF) represent the most widely cultivated, processed and consumed fruits throughout the world thanks to their pleasant taste, and they have become relevant components of the diet also for their acknowledged beneficial properties, such as antioxidative, anti-inflammatory, anti-infective, anti-cancer and neuroprotective [9,10,11,12,13,14].

Among CF, the most known include sweet orange (*Citrus sinensis* (L.) Osbeck), lemon (*Citrus limon* (L.) Osbeck), mandarin (*Citrus reticulata* Blanco), bergamot (*Citrus* × *bergamia* Risso & Poiteau), grapefruit (*Citrus paradisi* Macfad.), pomelo (*Citrus maxima* (Burm.) Merr.), lime (*Citrus aurantiifolia* (Christm.) Swingle) and citron (*Citrus medica* L.). Although part of CF by-products from food processing industry are recycled, a large quantity is released in the environment, causing pollution issues. Hence, these processing residues are considered as a waste to be disposed, rather than a valuable resource to be reused. However, the scientific community has recently supported the reusing/recycling of CF as well as other fruit or vegetable waste products [15], even if, often, the use of by-products is limited due to the still scarce understanding of their nutritional and economic value.

This review gathers the most recent evidence on the pharmacological potential of CF waste products, supporting their antimicrobial, anti-inflammatory, anti-oxidant, anti-cancer and neuroprotective activities in several in vitro and in vivo models, thus suggesting their use as nutraceuticals.

## 2. *Citrus* Fruits

The genus *Citrus* L. belongs to the Rutaceae family, subfamily Aurantioideae [16]. The fruits of the several species of this genus are cultivated all over the world, and their annual production has been growing considerably and rapidly due to rising consumers’ demand. They are mainly grown in tropical and sub-tropical areas of South-East Asia, in the Mediterranean countries of Europe and North Africa, in America, South Africa and Australia. In particular, global CF production for 2020/21 amounts to 98 million tons, of which the main producing countries are China, Brazil, European Union and United States (USA). Around 11 million tons of CF are exported worldwide, of which more than 40% being oranges and almost 30% being mandarins. The first CF exporting country is South Africa, followed by Turkey, Egypt and USA. In the last two years, world production of oranges has been estimated at 48.6 million tons, while that of mandarin has increased up to 33.3 million tons for 2020/21, as well as grapefruit and lemon production which grew up to 6.7 and 8.4 million tons, respectively [17].

The phylogenetic relationships within the genus *Citrus* L., and/or between this and related genera, are based on markers obtained by the use of modern molecular biology techniques: isozymes, Restriction Fragment Length Polymorphisms (RFLPs), Sample Sequence Repeats (SSRs), Inter-Sample Sequence Repeats (ISSRs), Random Amplification of Polymorphic DNA (RAPD), cpDNA sequence and Amplified Fragment Length Polymorphism (AFLP). The hybrid origin of many “species” has been demonstrated within the genus *Citrus* L.: Mabberley’s classification, clarifying the phylogenetic relationships among the three true species and their involvement in different hybrids, states that in the group of edible *Citrus* fruits, there are only three wild species: *Citrus medica* L. citron, *Citrus maxima* (Burm.) Merril pomelo and *Citrus reticulata* Blanco mandarin. Each of these is involved in several hybrids.

*Citrus* plants are generally evergreen shrubs or small trees with very fragrant flowers, which prefer moderate climatic conditions with optimum temperatures of 20–35 °C. The fruit, which is closely related to a berry and develops from a syncarpous gynoecium with axile placentation, is a hesperidium of different shapes (e.g., round, oblong or elongated) with a diameter from 3.8 to 14.5 cm. The exocarp (flavedo), tissue with essential oil cavities, consists of small, dense collenchyma cells which contain chromoplasts. The ultrastructural study of fruit peel by means of scanning electron microscopy has clarified the schizolysigenous nature of the secretory tissues, where the essential oil is produced and stored [16]. The epidermis resembles a cobbled surface and consists of very small, thick-walled cells, containing chromoplasts and oil droplets. In addition, a few scattered stomata can be found in the epidermis. The mesocarp (albedo) is a tissue with a spongy nature, consisting of loosely connected colorless cells and numerous air spaces in it, hence conferring a white color to this part of the hesperidium. The endocarp is relatively thin and consists of very elongated, thick-walled cells that form a compact tissue. The stalked, spindle-shaped juice vesicles, which fill the locules when the fruit ripens, develop from the cells of the inner epidermis and subepidermal layers. Each juice vesicle is covered externally by a layer of elongated cells, and encloses very large and extremely thin-walled juice cells containing acids, oils and sugars, which give the characteristic flavor to fruit. An increase in total sugar and in fruit dimension, as well as a decrease in acidity and change in peel color, vary according to the stage of maturation of each CF. Essential oil cavities occur characteristically also in the mesophyll of the leaves and in the petiole [16].

## 3. *Citrus* Waste

Almost a fifth of the total *Citrus* cultivars are subjected to industrial processes [18], mainly for the production of their juices that represent the most consumed fruit juices worldwide, thus generating a large amount of processing waste (about 120 million tons per year) [15,19]. However, this industrial process exploits only the 45% of the total fruit weight, whereas the rest, such as peel (flavedo; 27%), pulp (albedo and endocarp; 26%) and seeds (2%), constitutes a disposal rest [20]. Moreover, the whole fruits that do not satisfy quality requirements, hence being discarded, increase these amounts. In parallel, pruning is a practice often applied to remove tree branches or improve the quality of the fruit, implying the production of large quantities of leaves [20], a residue that increases the already large amount of *Citrus* waste. Especially in developing countries, this is often thrown into landfills or rivers, causing environmental pollution or contamination of water, with depletion of dissolved oxygen levels. The high rate of pollution given by *Citrus* waste depends to its easy fermentability, since it is abundant, chemically complex, biodegradable, thus requesting high oxygen demand. Generally, it has a low pH (3–4), with a high content of water (80–90%) and organic matter (95% of total residue) [21]. As another option, CF waste is disposed also through combustion to obtain thermal energy [22], a dangerous procedure that releases in the environment high levels of nitrogen, carbon and sulfur oxides, hence being no longer a suitable strategy for CF waste disposal. Therefore, in order to reduce environment pollution and gain profit to industries, several alternatives were proposed for better management of CF waste, such as the production of fortified animal feeds, the use of fiber-rich components in confectionery products, the extraction of macro- and micro-nutrients, as well as the production of organic fertilizers, bio-fuels, enzymes and ethanol, employed in the food, pharmaceutical, and cosmetic industries [5,19,23]. Indeed, the development of waste biorefineries in industrialized countries could provide significant economic (i.e., the recovery of energy and value-added products, land savings, new businesses and consequent job creation, landfill cost savings, etc.) and environmental benefits (i.e., greenhouse gas emissions reduction and savings of natural resources of land, soil and groundwater, energy, etc.) [24]. However, the processing of CF waste by biorefineries often requires certain costs. For example, it has been estimated a total cost to produce ethanol using 100,000 tons/years waste of 0.91 USD/L, assuming a cost of 10 USD/ton associated to handling and transportation to the biorefinery [25]. Anyway, due to its heterogeneity, CF waste is considered a valuable economic and renewable source for cosmetic (i.e., to obtain skin care products such as soaps, lotions, body sprays, essential oils), pharmaceutical and health (i.e., for the production of nutraceuticals, functional foods) industries [26]. Moreover, these re-use strategies could allow food industries to reduce the amount of waste and the costs of its disposal as organic matter, obtaining new commercial products (i.e., food packaging, production of antimicrobials, encapsulating agents, additives and prebiotics).

## 4. Composition of *Citrus* Waste

By-products from *Citrus* fruits, including exhausted peel, seeds, pressed pulp, secondary juice (obtained by pressing the residual pulp after the primary juice extraction) and leaves, are a source of polyphenols (i.e., flavonoids and phenolic acids), sugars (i.e., glucose, fructose and sucrose), dietary fibers (i.e., pectin and cellulose), proteins, lipids (i.e., linolenic, oleic, palmitic and stearic acids), organic acids (i.e., citric, malic and oxalic acids) carotenoids (i.e., carotene and lutein), vitamins (i.e., vitamin C and vitamin B complex) and monoterpenes (i.e., limonene and linalool) [27]. Molecular composition of each by-product may vary depending on the type of cultivar, the cultivation method, the harvesting time and the degree of ripeness of the fruit.

**Figure 1:**
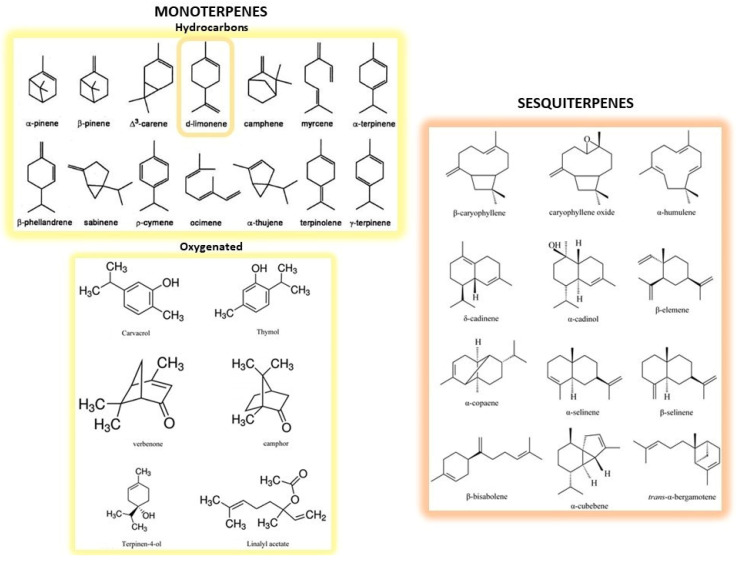
Structures of the main monoterpenes and sesquiterpenes present in CF peels. Monoterpenes represent the simplest terpenes, with two isoprene units and ten carbon atoms. They are divided into hydrocarbons (on the **upper left**) and oxygenated derivatives (on the **lower left**). Among the former, d-limonene (circled) is the main constituent of essential oils. Sesquiterpenes (on the **right**) are more complex terpenes possessing three isoprene units and fifteen carbon atoms.

CF peel represents almost 50% of the wet fruit mass after juice extraction [28], and is particularly high in fragrant compounds, dietary fibers, pectin, natural pigments as well as polyphenols [29]. CF peel is mainly employed for the extraction of essential oils (EOs), which are contained in the oil sacs of both peels and cuticles, although they can also be isolated from seeds or leaves in much lesser quantities. Chemical composition of EOs consists of monoterpenes and sesquiterpenes compounds (i.e., hydrocarbons with two or three isoprene units in their structure) and oxygenated derivatives (i.e., alcohols, ketones, aldehydes and esters; Figure 1). Limonene is the major constituent of EOs extracted from *Citrus* by-products, whereas β-pinene, sabinene and β-ocimene are characteristic of the EOs from *Citrus* leaves [30]. CF EOs have long been used as flavorings in preparation of food, cosmetic and pharmaceutical products and, more recently, have been re-evaluated for their health beneficial properties [31,32].

**Figure 2:**
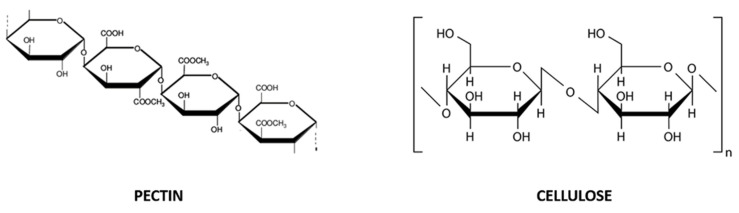
Chemical structure of pectin (D-galacturonic acid units linked together by α-1,4 glycosidic bonds; on the **left**) and cellulose (glucose units −300–3000 molecules- linked by a β-1,4 glycosidic bond; on the **right**), soluble and insoluble dietary fibers, respectively.

Exhausted CF peels are a source of pectin and dietary fibers, which are also present in juice and pulp [33]. Pectin is a complex polysaccharide composed of D-galacturonic acid units linked together by α-1,4 glycosidic bonds, partially esterified with methanol or acetic acid (Figure 2). It commonly exists in complex or insoluble forms, from white to light brown color and, being a naturally gelling agent, it is used as a thickener, emulsifier, texturizer and stabilizer in the preparation of confectionery, jams and jellies, as well as biodegradable products. Dietary fibers are non-starch polysaccharides including at least ten carbohydrate units (Figure 2), not easily digested nor absorbed in the intestine, and they can exist in soluble (i.e., gum, pectin and a part of cellulose) and insoluble forms (i.e., cellulose, hemicellulose and lignin) [33].

**Figure 3:**
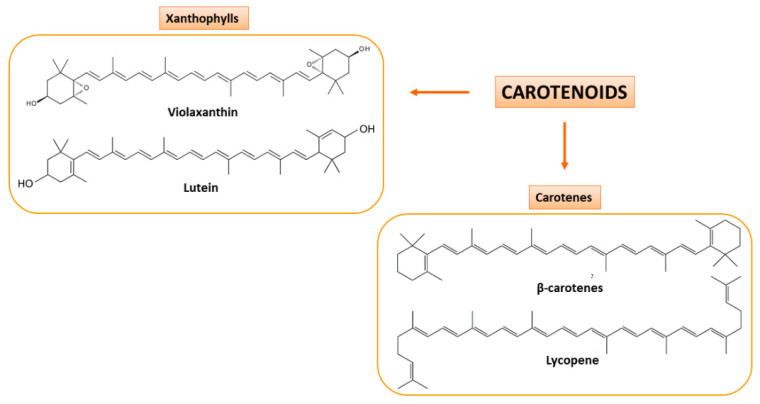
Carotenoids present in CF juices. Based on their chemical composition, they are classified as xanthophylls (oxygenated carotenoids; on the **left**) and carotenes (hydrocarbon carotenoids; on the **right**). Some examples of the two classes of carotenoids are here shown.

Secondary CF juices are a great source of carotenoids and flavonoids that are present also in peels. Carotenoids are pigments biosynthesized in different fruits and vegetables and can be subdivided into two classes: oxygenated carotenoids (or xanthophylls), such as lutein and violaxanthin, and hydrocarbon carotenoids (or carotenes), such as β-carotene and lycopene [34,35] (Figure 3). They are precursors of vitamin A, which is involved in epithelial tissues growth, strengthening of the immune system and promotes proper functioning of vision [36].

**Figure 4:**
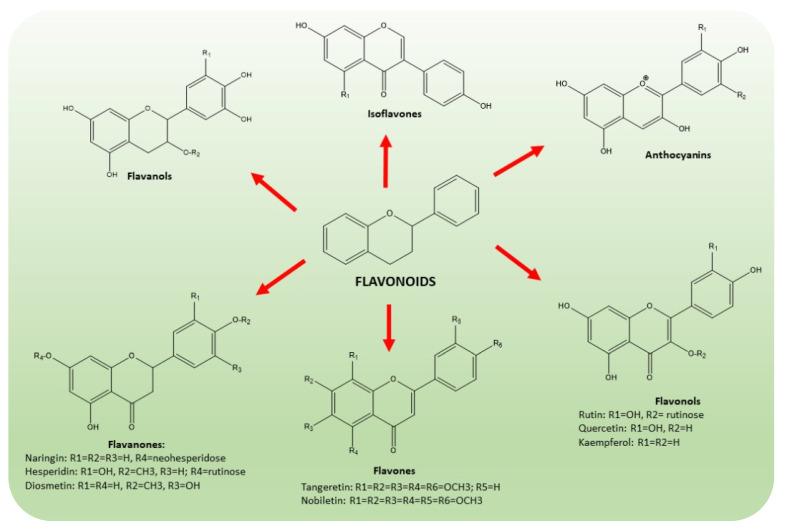
Chemical structures of the main classes of flavonoids present in CF, namely flavones, flavanones, flavonols, isoflavones, anthocyanidins and flavanols. The common scaffold of flavonoid class consists of a benzene ring joined to a benzo-γ-pyrone moiety.

Flavonoids are a wide class of secondary metabolites, synthesized by plants to protect against ultraviolet radiation or pathogenic injuries. According to their structure (Figure 4), they are subdivided into six groups, which are flavones, flavanones, flavonols, isoflavones, anthocyanidins and flavanols [37,38,39,40]. *Citrus* flavonoids have been extensively studied for their anti-cancer [41], anti-inflammatory [42] and neuroprotective activities [43]. In particular, naringin, hesperidin, hesperetin, neohesperedin, narirutin and rutin were quantified as the main flavanones of “satsuma mandarin” juice processing waste [44].

**Figure 5:**
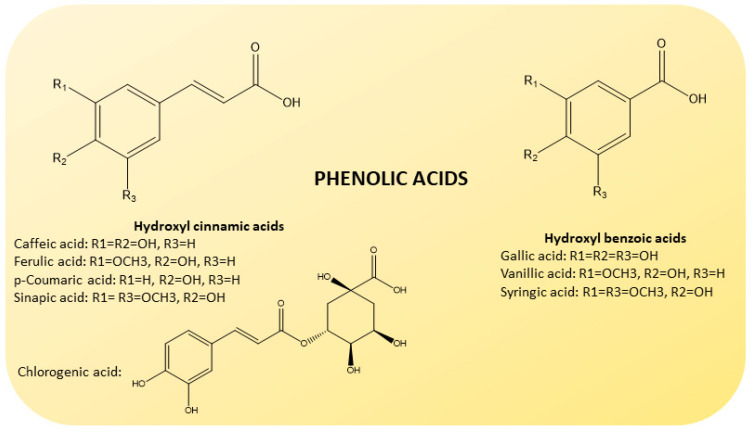
Chemical structure of phenolic acids present in CF juices. Based on their origin, they are divided into hydroxybenzoic (gallic, vanillic and syringic acids; on the **right**) and hydroxycinnamic (caffeic, ferulic, p-coumaric, sinapic and chlorogenic acids; on the **left**) acids.

Phenolic acids are also present in some amount [44] (Figure 5). They are divided into hydroxybenzoic (gallic, vanillic and syringic acids) and hydroxycinnamic acids (caffeic, ferulic, p-coumaric and sinapic acids), known to possess high levels of free radical scavenging activity [45].

Seeds are isolated during the juice extraction, and are a useful source of oil, proteins, limonoids and phenolic compounds, in particular, the flavonoids eriocitrin and hesperidin [46].

## 5. *Citrus sinensis* (L.) Osbeck

*Citrus sinensis* (L.) Osbeck (sweet orange) is a small evergreen tree originating from Southern China and arrived in Europe between the sixteenth and seventeenth century. Its fruits are a rich source of vitamin C and secondary metabolites, such as flavonoids, carbohydrates, carbamates, alkylamines, carotenoids, volatile compounds and in small amounts of natural elements as calcium, potassium, sodium and magnesium [47]. Sweet orange is thought to be a cross between mandarin (*Citrus reticulata* Blanco) and pummelo (*Citrus maxima* L. Osbeck). *Citrus sinensis* (L.) Osbeck varieties differ in origin, taste and size. They include two blond ones, Valencia late and Washington navel, and three red or blood ones, Moro, Sanguinello and Tarocco, typical of Eastern Sicily (Italy), California and Spain, respectively.

Orange is widely processed to produce juices, candies and extracts for food industries. Orange juice (OJ) is among the most consumed *Citrus* juices in the world and was extensively studied for its health benefits. In particular, its antioxidant activity as well as that of its flavonoid-rich extract (OJe) was observed in both *cell-free* and *cell-based* experimental models [48,49,50,51]. Moreover, OJe showed anti-inflammatory [52,53], anti-epileptic [54] and anti-obesity [55] effects in in vivo models, while the orange EO was suggested to possess interesting anti-anxiety properties [56].

Due to its widespread consumption, a large amount of waste is generated from orange processing industry, accounting approximately for 50% of the total CF one [19]. Recently, orange by-products have been claimed to represent a rich source of nutraceuticals, according to their characterization [57,58]. In these regards, an ethyl acetate extract of Newhall orange peel (*C. sinensis* (L.) Osbeck cultivar Newhall), one of the greatest sources of waste-producing CF in China, proved to be a potent antioxidant, antibacterial agent and tyrosinase inhibitor [59]. The rich phytochemical constituents of *C. sinensis* peel extract, which include phenolic and flavonoid compounds, appeared also to contribute to its antioxidant potential [60]. In addition, pectic oligosaccharides recovered from orange peel waste showed prebiotic effects on bifidobacteria and lactobacilli levels in in vitro model of fermentation using human fecal inocula [61].

Discarded seeds of Hamlin, Natal, Perario and Valencia orange varieties are promising sources of essential oils, and their chemical composition accounts for a high content of carotenoids, phenolic compounds, tocopherols, and phytosterols, which play a relevant role in the free radical scavenging capacity of this by-product [62]. Moreover, it was demonstrated that orange seed oil and non-oil extracts possess interesting antibacterial and antifungal properties, other than antioxidant, helpful for the development of antimicrobial agents [63].

Besides *Citrus* processing, sweet orange pulp may be re-evaluated for its content of valuable compounds, such as phenolics and especially hesperidin [64]. In this line, a red orange and lemon extract produced from pulp processing waste demonstrated a good antiallergic activity, mainly reducing basophils activation and degranulation, as well as the pro-inflammatory mediators release induced by allergic stimuli [65]. Analogously, different by-products derived from the industrial extraction of orange juice showed anti-inflammatory effect in mice with dextran sulfate sodium-induced colitis [66].

In addition, a clear example of a possible application of the circular economy in the food system was the production of a fiber-rich flour from an orange juice by-product, which was used to obtain a fortified food to increase dietary intake of fiber, minerals and phenolic compounds [67].

Therefore, despite the high levels of waste in the food supply chain, the enormous availability of orange juice, peel, pulp and seeds could be helpful to recover valuable compounds in line with green economy.

## 6. *Citrus aurantiifolia* (Christm.) Swingle

*Citrus aurantiifolia* (Christm.) Swingle (lime) is a tree belonging to the tropical Northern Indian area. It was brought by Arabs into Middle East, Northern Africa and Mediterranean Europe, and then by Spanish expeditions into Central and South America to be so far cultivated. It seems to come from a cross between *C. medica* and *C. micrantha*. The fruit has a greenish-yellow color and a sour and bitter taste unlike sweet lime, that is known as *Citrus limetta*, whose flavonoid profile and antioxidant activity were determined [68].

Lime peels represent a by-product of the juice manufacturing industry and are used to extract EOs. Besides exhibiting potent free radical scavenging activity, this lime EO exerts protective effects against lipid-induced hyperlipidemia in a rat model by improving cholesterol, triglyceride, alanine aminotransferase and aspartate transaminase levels [69]. Furthermore, lime EO was able to alleviate inflammation both in vitro and in vivo models. Indeed, pre-treatment with lime EO inhibited the production of pro-inflammatory cytokines (i.e., tumor necrosis factor-TNF-α, interleukin-IL-6, IL-1β) in lipopolysaccharide (LPS)-stressed macrophages as well as the production of reactive oxygen species (ROS) induced by H_2_O_2_. Topical application of lime EO led also to reduction of the 12-O-tetradecanoylphorbol-13-acetate (TPA)-induced mouse ear inflammation [70]. Finally, the anti-malarial capability of a lime peel extract has been associated to the antioxidant and anti-inflammatory properties of its flavonoid constituents both in LPS-stimulated macrophage cells and in *Plasmodium berghei*-infected mice [71]. Regarding seed waste, the *C. aurantiifolia* Swingle seeds extract exerts cytotoxic effect against L5178Y human lymphoma cell line [72], while an extract obtained by lime leaves has demonstrated in vitro antibacterial effect against some Gram-positive (*Bacillus subtilis*) and Gram-negative (*Salmonella* spp., *Escherichia coli*, *Streptococcus faecalis*, *Staphylococcus aureus*) bacteria [73]. Moreover, the recycling *C. limetta* pulp exerts antimicrobial activity against both Gram-positive and Gram-negative bacteria, suggesting that it can be considered a useful resource for the bio-economy [74].

## 7. *Citrus maxima* (Burm.) Merr.

*Citrus maxima* (Burm.) Merr. fruit, commonly known as “pomelo” or “pummelo” or “shaddock”, is a parent of the grapefruit (*C. sinensis* × *C. maxima*), and its cultivar probably originated from Thailand. To date, it is spread in South-East Asia and China and, limitedly, in Florida, California and Hawaii. Fruits are the largest CF in the world and, depending on the cultivar, it can show peculiar phenotypic characteristics, such as being with seeds or seedless, yellow, red or colorless. Generally, it is consumed fresh or processed into juice, but it has been used in folk medicine to counteract fatigue, lack of vitality and energy, wounds, acne, osteoarthritis or minor skin disorders. The pomelo processing industry allow recovering valuable products from the wide residual waste content it generates.

*C. maxima* peel is frequently discarded after fruit consumption, meaning high economic and environmental costs for society. The health and therapeutic potential of pomelo peel extracts were recently summarized, highlighting hypolipidemic, hypoglycemic, antimicrobial, antioxidant, anti-inflammatory and anticancer effects [75]. Pomelo peel extract, along with its four major coumarins (auraptene, marmin, isoauraptene, meranzin hydrate), exerts anti-inflammatory effects in LPS-stressed RAW 264.7 cells, reducing the release of pro-inflammatory cytokines IL-1β, prostaglandin 2, and TNF- α [75,76]. In addition, they are also able to alleviate xylene-induced ear edema and carrageenan-induced paw edema in mice [76]. In addition, *C. maxima* waste (flesh, peels, carpel and the essential oil) can counteract metabolic disorders such as obesity and hyperlipidemia. Indeed, experimental data revealed their anti-lipogenic potential in Wistar rats by involving AMPK-SREBP-PPARS pathway [77]. Moreover, representative pomelo phytonutrients, such as limonene, γ-terpinene, p-synephrine, β-sitosterol and hesperidin, down-regulate the expression of key enzymes in *de novo* biosynthesis of cholesterol and triacylglycerides in HepG2 cells [77]. EOs isolated from pruning of Mato Peiyu pomelo leaves are mostly composed of citronellal and citronellol (>50%), which are hence the main players of EO’s antimicrobial, antioxidant, anti-inflammatory and anti-tyrosinase activities [78]. Moreover, the EO of *C. maxima* leaves proved to be a stronger antimicrobial agent than the EOs of *C. sinensis* and *C. aurantifolia* leaves [30].

## 8. *Citrus reticulata* Blanco and Its Hybrids

*Citrus reticulata* Blanco (mandarin) tree originated from South-East China, and after it was cultivated mainly in Italy and Spain. Mandarin group is phenotypically heterogeneous, including numerous species, hybrids and mutants cultivated throughout the world.

The peel of a Chinese variety of *C. reticulata* Blanco (Ponkan) is considered as waste material. However, a hydrodistillation method was employed to extract EO from Blanco peel, mainly constituted by limonene (89.31%), which exhibits antibacterial activity against *Cutibacterium acnes* and common microorganisms such as *S. aureus*, *B. subtilis* and *E. coli* [79]. In addition, besides the antimicrobial potential, EO from *C. reticulata* peel has been able to significantly reduce wound diameter in vivo, thus showing wound healing property [80]. Mandarin peels contain also a high total flavonoid content compared to lemon and grapefruit peels, and have been demonstrated to possess moderate cytotoxic activity against the HL-60 cell line, an experimental model of acute myeloid leukemia [81]. Moreover, mandarin peel, as well as lemon and grapefruit ones, showed immunomodulatory activity, since it increased the proliferation of mouse splenocytes and antigenotoxic effect, through reduction of cisplatin-induced chromosomal aberrations in the same experimental model [81]. Interestingly, a flavonoid-rich extract of *C. reticulata* juice showed promising anti-cancer activity in anaplastic thyroid carcinoma cell lines [82], as well as proved to be a strong neuroprotective agent in an in vitro model of Parkinson’s disease [83].

Despite being a wide source of polysaccharides, the peel of a hybrid of mandarin and orange typical of South Korea is commonly discarded. This waste product can be re-considered for its anticancer properties. Indeed, it showed anti-angiogenesis effects by counteracting tube formation of HUVECs endothelial cell line and cell cancer migration through downregulation of matrix metalloproteinase-9 (MMP-9) expression in MDA-MB-231 cells, thus revealing a potential therapeutic agent in triple-negative breast cancer metastasis [84].

Many by-products of “satsuma mandarin” come from the food industry and are a source of agricultural waste. High polyphenol content of fermented *Citrus* flesh and peel by-products is responsible for the potent antioxidant and antibacterial activities against *Listeria monocytogenes* and *E. coli* [85]. Another fermented extract prepared from “satsuma mandarin” peel, after juice processing, arrested the growth of human pancreatic cancer cells, through the induction of apoptotic mechanism, and blocked their migration via the activation of intracellular signaling pathways, where MKK3/6 and P38 proteins are involved. In addition, this extract showed anticancer effects in a xenograft experimental model. A more in-depth analysis, performed *in silico*, demonstrated that the flavonoids naringenin and hesperetin were associated to the anticancer properties of this fermented extract [86]. Previously, another flavonoid, quercetagetin, isolated by methanolic extract of “satsuma mandarin” peel powder, has been shown as radical scavenger capable to reduce ROS levels and to protect against H_2_O_2_-induced DNA damage in Vero cells [87].

During the juice extraction process *Citrus junos* Siebold ex Tanaka (yuzu, *C. ichangensis* × *C. reticulata* hybrid) seeds are discarded. This waste product contains high amounts of limonoid aglycones, glycosides and oils which showed to possess antioxidant capability and radical scavenging activities [88].

Increasing demand for mandarin juice generates more and more waste from processing industries. Satsuma mandarin waste (i.e., albedo, flavedo, pulp residues and carpellary membranes) is rich in extractable and non-extractable hesperidin and eriocitrin and non-extractable gallic acid. The drying procedure of this waste can quantitatively affect the composition of the extracts, being a crucial step to obtain a valuable product from these residues [89].

Peel, pulp and seed extracts from two mandarin varieties, namely Phlegraean mandarin and clementine were compared to evaluate functional components of each cultivar. Phlegraean mandarin peel and seeds possess the greatest variety of polyphenols. Vitamin C is abundant in Phlegraean mandarin seeds while clementine peel and pulp are rich of carotenoids. Antioxidant capacity of the peel and seeds of Phlegraean mandarin, clementine peel was observed, thus highlighting relevant properties belonging to parts of the fruit generally considered only waste [90].

Murcott mandarin (hybrid of *C. reticulata* × *C. sinensis*) leaf fraction seems to induce gastroprotective and anti-ulcerogenic effects against alcohol-induced gastric ulcers in rats thanks to its known anxiolytic, anti-inflammatory, antioxidant and anti-apoptotic properties [91].

EOs from the leaf and fruit peel of *C. reticulata* Blanco cultivar Santra reduce LPS-stimulated TNF-α and NO levels in RAW 264.7 murine macrophage cell culture, exhibiting anti-inflammatory properties [92].

Finally, clementine by-products (peels and leaves) could represent a promising source of functional foods destinated to the prevention of oxidative stress- related diseases such as type 2 diabetes and obesity, thanks to their anti-oxidant properties [93].

## 9. *Citrus paradisi* Macfad.

*Citrus paradisi* Macfad. (grapefruit) is probably a natural hybrid between *C. maxima* × *C. sinensis* that was discovered in the Caribbean and afterwards brought to Europe. *C. paradisi* fruits can be divided into two groups, depending on the presence (“Rose Pink” and “Ruby Red”) or absence (“White Marsh”) of lycopene in the fruit flesh [94]. Grapefruit juice is acknowledged for its noteworthy pharmacokinetic interactions [95], as well as for its anti-inflammatory and antioxidant properties [96].

Among wasted parts of *C. paradisi* fruit, the peel had a higher content of phenolic compound, flavonoids, vitamin C, and hence a higher antioxidant activity, than pulp and seeds. In addition, peels of grapefruits possess the highest total phenolic content compared to lemon and orange peels [97]. The oil obtained from *C. paradisi* fresh leaves is mainly characterized by limonene, while the major component of fresh peel oil is β-phellandrene. Each oil, depending on its origin (i.e., peel or leaves), possesses a characteristic odor, which make it useful flavoring agent. In addition, both oils reduce the egg albumin-induced oedema size in rats, suggesting their anti-inflammatory capacity [98]. Given the above-mentioned qualities of grapefruit waste, its revalorization can be beneficial to several fields. Indeed, *C. paradisi* peel is mainly utilized as biosorbent, while the extracted oil is applied in aromatherapy and its main component, limonene, is used as antimicrobial or in cosmetics sector. Grapefruit pectin is exploited for bioethanol production or as biosorbent, and peel phenolics biotransformation is promoted [99].

## 10. *Citrus limon* (L.) Osbeck

*Citrus limon* (L.) Osbeck (lemon) is reported to originate from South-East Asia. Then, Arabs brought this *Citrus* in Europe in twelfth century. It is known to be a hybrid between *C. medica* × *C. reticulata* × *C. maxima*. The juice is known to possess several beneficial activities, among which anti-bacterial [100] and antioxidant ones [101].

A reuse of *C. limon* waste as a possible resource has already been considered in the past. In this field, the waste stream of lemon peels has been shown to exert plasma and liver cholesterol-lowering effect in hybrid F1B hamsters [102], while a mixture of pectooligosaccharides obtained from lemon peel waste showed prebiotic effects on the microbiota of elderly donors, via modulation of beneficial bacteria levels (*Faecalibacterium prausnitzii* and *Blautia*) and production of elevated alpha diversity values [103]. Lemon pomace waste and pulp residue were also shown to possess high phenolic and flavonoid content that are responsible for their relevant antioxidant activity [104,105]. Silver nanoparticles synthesized from *C. limon* peels waste revealed antimicrobial activity against most human pathogenic bacteria (*Acinetobacter baumannii*, *Salmonella typhimurium*, *E. coli*, *Pseudomonas aeruginosa*, *S. aureus* and *Proteus vulgaris*) [106], as well as an extract of yellow lemon peel against *Klebsiella pneumoniae* [107], thus representing a potential remedy to combat certain multidrug resistant microorganisms. Moreover, the aforementioned silver nanoparticles induced in vitro have a cytotoxic effect in some types of human tumors, including breast and colon cancer [106]. *C. limon* pectic polymer derived from the industrial residue of *Citrus* juice extraction via hydrodynamic cavitation process exerts significant neuroprotective effects against H_2_O_2_-induced damage on SH-SY5Y cells, by preserving cellular viability and morphology as well as limiting reactive oxygen species (ROS) production and related mitochondrial damage [108].

Recently, EOs from lemon, mandarin and grapefruit seeds discarded by an agro-alimentary industry were studied to evaluate their nutritional and biological properties. Noteworthy, even if to a different extent, all three oils possess antioxidant, antifungal and antitumor properties, inhibitory effect on the mushroom tyrosinase activity, as well as and antiproliferative effect on murine B16F10 melanoma cells [46]. EOs isolated from lemon leaves, rich in limonene, citronellal and citronellol, possess antibacterial activity against Gram-negative (*Salmonella typhimurium*, *P. aeruginosa* and *E. coli*) and Gram-positive bacterial strains (*Staphylococcus epidermidis* and two *S. aureus* strains), as well as insecticidal potential [109,110].

## 11. *Citrus medica* L. and Its By-Products

*Citrus medica* L. (citron) is thought to come from Indian region and is one of the first species of *Citrus* arrived in the Mediterranean basin, since Greek and Latin sources confirmed its presence in their diets. Indeed, its name originates from the “Media” term with which referred to the Persian area, where this fruit was discovered by the Greek during Alexander the Great’s Empire. Along with *C. maxima* and *C. reticulata*, *C. medica* represents one of the three ancestral taxa from which *Citrus* fruit recovers its origin.

Despite representing a rich resource of antioxidants and antibacterial compounds, the citron peel is usually discarded as waste. The EO extracted from it showed antibacterial activity against *S. aureus* e *E. coli* and an interesting radical scavenging activity [111], typical effects of potential additive useful for the food industry.

## 12. *Citrus* × *bergamia* Risso & Poiteau and Its By-Products

*Citrus bergamia* Risso & Poiteau (bergamot) is a hybrid between citron and orange, which is itself a cross between pomelo and mandarin: *C. medica* L. × (*C. maxima* (Burm.) Merr. × *C. reticulata* Blanco). It seems to originate from various countries such as Canary Islands, Greece, Antilles and then arrived to the Spanish city “Berga”, from which the name “bergamot” is derived. It spontaneously grows along the Ionian coast of the Calabria region (Southern Italy) where meets its favorable microclimate. Three different cultivars are known: “Castagnaro”, “Femminello” and their hybrid, “Fantastico”. Bergamot is mainly known for bergamot essential oil (BEO) mostly used as fragrance of many perfumes, in cosmetic industry, in confectionary products and food industries. Moreover, BEO revealed antimicrobial activities [112], as well as an anti-cancer effect [113,114,115]. BEO is currently employed in the field of aromatherapy [13,116] and evaluated for its neuroprotective [117], anti-inflammatory and analgesic capabilities [118]. Recently, bergamot juice (BJ) has also been evaluated for its great pharmacological value. Indeed, it has been reported its antimicrobial activity against *Helicobacter pylori* [119], and its anticancer activity both in in vitro and in vivo models [120,121,122,123,124]. Given the high polyphenolic content of BJ [125], its flavonoid-rich fraction (BJe) was further studied for the hypolipemic and hypoglycemic activities [126], neuroprotective [127] as well as the anti-inflammatory potential [128]. In this field, BJe prevented both LPS- and β-amyloid-induced inflammatory response in THP-1 monocytes, by modulating NF-κB pathway via the AMPK/SIRT1 [129,130] axis and the MAPK/AP-1 pathways, respectively [131]. Moreover, BJe proved to be an effective anti-inflammatory product also in several in vivo models [132,133,134]. Interestingly, the chemical composition of BJ and its secondary juice, despite quantitative differences between them, are similar [120,121,124,125], thus suggesting the potential of this by-product in the pharmacological field.

Two flavonoid-rich extracts from bergamot peel, which represents the main agri-food waste (about 60%) of the processed bergamots, exerted protective effects against cell modifications induced on human endothelial cells (HUVECs) exposed to inflammatory cytokine tumor necrosis factor-α (TNF- α) [135]. In addition, bergamot peel extracts modulated intracellular levels of malondialdehyde/4-hydroxynonenal, glutathione and superoxide dismutase activity, and the activation of NF-κB in HUVEC cells, hence proving its high content of antioxidant and anti-inflammatory compounds [135]. Moreover, the albedo is a potentially recyclable waste of bergamot industries, since it contains the flavonoids brutieridin and melitidin, which demonstrated to be helpful in the hyperlipidemia management [136].

The bioactive compounds as well as the pharmacological properties of *Citrus* derivatives and by-products discussed in this review are summarized in Table 1.

## 13. Conclusions

Nowadays, a proper management of CF waste is crucial in order to enhance the sustainability of their cultivation. Indeed, a re-evaluation of CF by-products could reduce their accumulation in the environment, as well as allow the exploitation of their full potential. This because CF by-products are an economic and renewable source of high value-added compounds, hence researchers should follow a holistic approach to appreciate the whole CF, including its waste, employing better strategies for its recovery and valorization. In this regard, different sustainable and green methodologies for the effective reuse of waste have been recently developed. Protocols for the recovery of valuable compounds include different steps: (i) extraction (i.e., enzyme-assisted, ultrasound-assisted, microwave-assisted, supercritical fluid, pressurized liquid extractions); (ii) separation and purification (via filtration and chromatographic techniques); (iii) identification and characterization (through NMR, mass spectroscopy or chromatography); (iv) toxicological screening both in vitro and in vivo [26,46,64,72]. Alternatively, conversion of CF waste into biofuels can be performed through thermochemical and biochemical processes such as pyrolysis, thermolysis, gasification, combustion [19].

This review provides scientific bases for the employment of CF by-products as nutraceuticals.

The scenario regarding future research and innovative methods for CF waste recovery is ever growing, but a combined effort between industry and research will lay the foundations for a more efficient circular economical system to generate wealth from waste, since one man’s trash is another man’s treasure.

## Figures and Tables

**Table 1:** Main bioactive compounds contained in *Citrus* by-products and their pharmacological activities.

*Citrus* Fruits	Bioactive Compounds	Activity	References
*Citrus sinensis* (L.) Osbeck	Flavonoids (hesperidin, naringin); limonoids (limonene); carotenoids; phenolic compounds; tocopherols and phytosterols; fiber, minerals and phenolic compounds; pectin; vitamins	Antibacterial and antifungal; antioxidant and anti-inflammatory; anti-cancer; antiallergic; anti-epileptic; anti-obesity; anti-anxiety; prebiotic	[48,49,50,51,52,53,54,55,56,59,60,61,62,63,64,65,66,67,137,138,139,140]
*Citrus aurantiifolia* (Christm.) Swingle	Flavonoids (hesperidin, naringin); tannins; alkaloids; polysaccharides; vitamins; minerals	Antioxidant and anti-inflammatory; anti-cancer	[68,69,70,71,72,73,141]
*Citrus maxima* (Burm.) Merr.	Flavonoids (hesperidin); coumarins (auraptene, marmin, isoauraptene, meranzin hydrate); limonoids (limonene, γ-terpinene, p-synephrine), phytosterol (β-sitosterol); citronellal and citronellol; polysaccharides; vitamins; minerals	Hypolipidemic; hypoglycemic; antimicrobial; antioxidant; anti-inflammatory; anti-cancer; anti-lipogenic	[30,75,76,77,78]
*Citrus reticulata* Blanco	Flavonoids (naringin, hesperidin, eriocitrin; quercetagetin); tannins; limonoids (limonene; β-pinene; sabinene); carotenoids; phenolic compounds; vitamins; pectins, polysaccharides; minerals	Immunomodulatory; antioxidant; anti-inflammatory; anticancer; antibacterial; antigenotoxic; anthelminthic; gastroprotective; anti-ulcerogenic	[79,80,81,82,83,84,85,86,87,88,89,90,91,92,93,142,143]
*Citrus paradisi* Macfad.	Flavonoids (naringin); limonoids (limonene; β-pinene); phenolic compounds; vitamins; pectins; minerals	Antioxidant; anti-inflammatory; anticancer; antibacterial	[96,97,98,99,137,144]
*Citrus limon* (L.) Osbeck	Flavonoids; limonoids (limonene, γ-terpinene, β-pinene, O-cymene, citral); 8-geranyloxypsolaren; 5-geranyloxy-7-methoxycoumarin; phloroglucinol 1-β-D-glucopyranoside (phlorin); phenolic acids; vitamins; pectins; minerals	Anticholesterolemic; antioxidant; antibacterial; neuroprotective; antitumor; prebiotic	[46,100,101,102,103,104,105,106,107,108,109,110,145,146]
*Citrus medica* L.	Flavonoids; phenolic acids; vitamins; pectins; minerals	Antibacterial; antiulcer	[111,147]
*Citrus* × *bergamia* Risso & Poiteau	Flavonoids (lucenin-2, vicenin-2, eriocitrin, neoeriocitrin, naringenin, hesperigin, neohesperidin, brutieridin, melitidin) furancoumarins (epoxybergamottin, bergamottin); limonoids; vitamins; pectins; minerals	Antibacterial; anti-inflammatory; antioxidant; anticancer; anti-lipoperoxidation; antidiabetic; prevention of cardiovascular diseases, anticholesterolemic; neuroprotective	[112,113,114,115,116,117,118,119,120,121,122,123,124,125,126,127,128,129,130,131,132,133,134,135,136,148,149]

## Data Availability

Not applicable.
